# 

**Published:** 1903-02-15

**Authors:** 


### CONTENTS.

The Predisposing Factor in Dental Caries, *By Edward C. Kirk, D.D.S.* 55

The Relations of the Board of Dental Examiners to the Profession, - - *By H. C. Brown, D.D.S.* 73

The International Dental Federation, *By H. A. Smith, D.D.S.* 81

Obtunding Sensitive Dentin with Cataphoresis, *By Weston A. Price, D.D.S,* 83

The Preliminary Education of the Dentist, *By Dr. C. M. Wright, D.D.S.* 87

Preliminary Education and College Training, *By Dr. Maurice Roy* 90

The Dental Curriculum of Sweden, *By Prof. L. Lindstroem* 97

Editorial, - - - - - - - - - - 100

Announcements, - - - - - - - - 106

Indents, - - - - - - - - - - 107

